# Fixed-dose streptozotocin combined with high-fat diet induces a stable type 2 diabetes-like phenotype in C57BL/6J mice

**DOI:** 10.1242/dmm.053071

**Published:** 2026-09-17

**Authors:** Claes Fryklund, Christian Simonsson, Alexandra Hellberg, Josefin Malmberg, Karin G. Stenkula, Maria Swanberg

**Affiliations:** Department of Experimental Medical Science, Lund University, 22100 Lund, Sweden

**Keywords:** Type 2 diabetes, Streptozotocin, High-fat diet, Insulin resistance, Hyperglycaemia

## Abstract

High-fat diet (HFD) combined with streptozotocin (STZ) is widely used to model type 2 diabetes (T2D) in rodents, but is often associated with high mortality, non-responders and inconsistent outcomes. STZ is conventionally administered using body weight-adjusted dosing (mg/kg), despite evidence that heavier animals, including HFD-fed mice, exhibit more severe glycaemic responses. Here, we performed metabolic phenotyping in chow- and HFD-fed C57BL/6J mice treated with low or high fixed doses (mg instead of mg/kg) of anomer-equilibrated STZ. HFD combined with low-dose STZ induced a stable T2D-like phenotype characterized by sustained obesity, moderate hyperglycaemia, insulin resistance and partial β-cell loss, with low inter-individual variability. In contrast, high-dose STZ induced a type 1 diabetes-like phenotype, with extensive β-cell loss. A semi-mechanistic mathematical model was developed and validated against independent experimental data, reproducing the observed dynamics of fasting glucose in response to fixed-dose STZ. The model further predicted that weight-adjusted (mg/kg) dosing could introduce variability in glycaemic responses, particularly in HFD-fed mice. Together, these results demonstrate that fixed-dose, anomer-equilibrated STZ induces a stable T2D-like phenotype, providing an alternative to conventional weight-adjusted dosing in HFD-fed mice.

## Teaser

**Summary:** Longitudinal metabolic phenotyping identifies a fixed-dose streptozotocin protocol that produces a stable type 2 diabetes-like phenotype in high-fat-diet-fed mice.

## INTRODUCTION

Type 2 diabetes (T2D) is a major global health problem, with recent estimates indicating that ∼500-800 million people are affected (NCD Risk Factor Collaboration, 2024; GBD Diabetes Collaborators, 2023). The disease substantially increases the risk of cardiovascular complications, including stroke and coronary heart disease, and was ranked as the eighth leading cause of death globally in 2019 (Emerging Risk Factors Collaboration et al., 2010; GBD Diseases Injuries Collaborators, 2020). T2D is a complex, polygenic disease arising from the interplay of genetic, epigenetic and environmental factors, with lifestyle factors such as physical activity and energy intake playing central roles (Prasad and Groop, 2015). Unlike type 1 diabetes (T1D), which is mainly driven by autoimmune β-cell destruction leading to insulin deficiency, T2D pathogenesis is highly heterogeneous and involves impairments in pathways related to β-cell function, insulin action, incretin signalling and adipose tissue distribution (Pearson, 2019). This heterogeneity has motivated the use of experimental mouse models that capture distinct aspects and stages of T2D pathology, from early insulin resistance and hyperinsulinemia (prediabetes) to late-stage β-cell dysfunction and hyperglycaemia. Among the most commonly used models are diet-induced obesity via high-fat diet (HFD), representing insulin resistance and a prediabetic phenotype; genetically modified strains (e.g. *ob/ob*, *db/db*), representing T2D-like phenotypes with both insulin resistance and hyperglycaemia; and toxin-induced β-cell depletion using streptozotocin (STZ), where high-dose protocols are used to induce an insulin-deficient, T1D-like phenotype (Singh et al., 2024).

C57BL/6J mice fed HFD exhibit insulin resistance, obesity and mild hyperglycaemia, representing a prediabetic state of T2D (The Jackson Laboratory, ‘Phenotype Information for B6 DIO’, accessed 20 July 2026). These mice resist progression to overt T2D by maintaining near-normal glucose through compensatory β-cell expansion and enhanced insulin secretion (Mosser et al., 2015; Kleinert et al., 2018). To induce overt T2D in C57BL/6J mice, the β-cell toxin STZ is often administered in combination with HFD feeding (Gilbert et al., 2011). STZ is taken up by pancreatic β-cells via GLUT2 transporters, where it causes DNA damage and β-cell death, leading to sustained hyperglycaemia (Wang and Gleichmann, 1998). STZ has historically been used primarily to model insulin-deficient, T1D-like diabetes in rodents, but more recent protocols have combined lower-dose STZ with HFD feeding to generate T2D-like phenotypes, with both insulin resistance and partial β-cell dysfunction (Gilbert et al., 2011; Furman, 2021).

The combination of repeated low-dose STZ injections after a few to several weeks of HFD feeding is a common approach to model T2D. However, there is no fully standardized protocol, and large variations in diet duration, STZ dosage and injection frequency likely contribute to inconsistent outcomes, including high mortality and non-responsive animals (Racine et al., 2024; Parilla et al., 2018; Karuna et al., 2025; Zheng et al., 2026; Deng et al., 2025; Goyal et al., 2016). Moreover, variability in response to STZ may arise from different protocols for STZ preparation, leading to differences in α/β-anomeric composition. As soon as STZ is dissolved, the more toxic α-anomer gradually converts to the less toxic β-anomer, a process that requires at least 2 h to reach an α/β-anomeric equilibrium (de la Garza-Rodea et al., 2010). Immediate injection of STZ after dissolution has long been recommended practice owing to presumed instability, but studies have demonstrated that STZ remains stable and biologically active for several days when stored cold and dark in acidic buffer (Povoski et al., 1993; de la Garza-Rodea et al., 2010). Recent studies recommend α/β-anomer equilibration (≥2 h) for improved reproducibility and reduced mortality (de la Garza-Rodea et al., 2010; Kamli-Salino et al., 2023).

One largely unexplored area in STZ-based models concerns the dosing strategy, as STZ has historically been administered using body weight-adjusted (mg/kg) dosing rather than fixed dosing (mg). Previous studies in rodents have shown that body weight-adjusted dosing results in more severe hyperglycaemia in heavier animals (Masiello et al., 1975; Gilbert et al., 2011). HFD-fed mice present a particular challenge owing to their highly variable weight gain, with their weight typically ranging ∼10–50% above that of chow-fed controls. STZ is hydrophilic and mainly distributes in lean body mass, with limited distribution into adipose tissue (Lenzen, 2008; Gouju and Legeay, 2023). Because HFD-induced weight gain is largely caused by fat accumulation, using total body weight may overestimate the relevant distribution space for STZ in obese mice. Body weight-adjusted dosing may, therefore, give obese mice a higher absolute STZ dose relative to lean body mass, without a proportional increase in the compartment relevant for STZ distribution. This may increase the risk of overdosing obese mice, while leaner mice may receive insufficient STZ exposure and remain non-responsive. We therefore hypothesized that fixed (non-weight-adjusted) STZ dosing would lead to more uniform β-cell toxicity across HFD-fed mice regardless of individual weight gain, improving reproducibility while minimizing non-responders and mortality. To test this hypothesis, we examined whether HFD combined with fixed, anomer-equilibrated STZ would induce a stable T2D-like phenotype, using longitudinal measures of glucose homeostasis, insulin resistance, β-cell function and pancreatic histology. The results were integrated in a systems biology mathematical model that predicted diet, STZ and time-dependent responses, and was validated in independent *in vivo* data.

This study provides important information for researchers using STZ-based diabetes models, particularly in combination with HFD feeding, where mice often show substantial variability in weight gain. The study design is relevant to T2D models as well as more severe insulin-deficient, T1D-like phenotypes, and proposes a fixed-dose STZ protocol to produce a reproducible T2D-like phenotype with low inter-mouse variability.

## RESULTS

### HFD combined with a fixed STZ dose mimics T2D pathology with sustained obesity and moderate hyperglycaemia

Body weight was monitored because HFD induces weight gain and STZ is known to cause weight loss (see Fig. 1 for study outline). Before STZ treatment (week 6), the HFD groups had, as expected, significantly higher body weights than those of the chow-fed groups (*P*<0.001, unpaired two-tailed Student's *t*-test) (Fig. 2A,B). At endpoint measurement (week 24), there was a significant main effect of STZ and diet, and a significant interacting effect of diet and STZ on body weight (*P*<0.001). Post hoc analysis revealed that HFD-STZ^low^ was the only STZ-treated group that continued to gain weight and remained obese, being ∼30% heavier than the control group at endpoint measurement (Fig. 2A,B). All other STZ-treated groups, including HFD-STZ^high^, stabilized at body weights slightly below those of chow-fed controls. Likewise, adiposity, measured as epididymal and inguinal depot weight, was significantly increased only in the HFD and HFD-STZ^low^ groups (Fig. S1A,B).

**Fig. 1.**
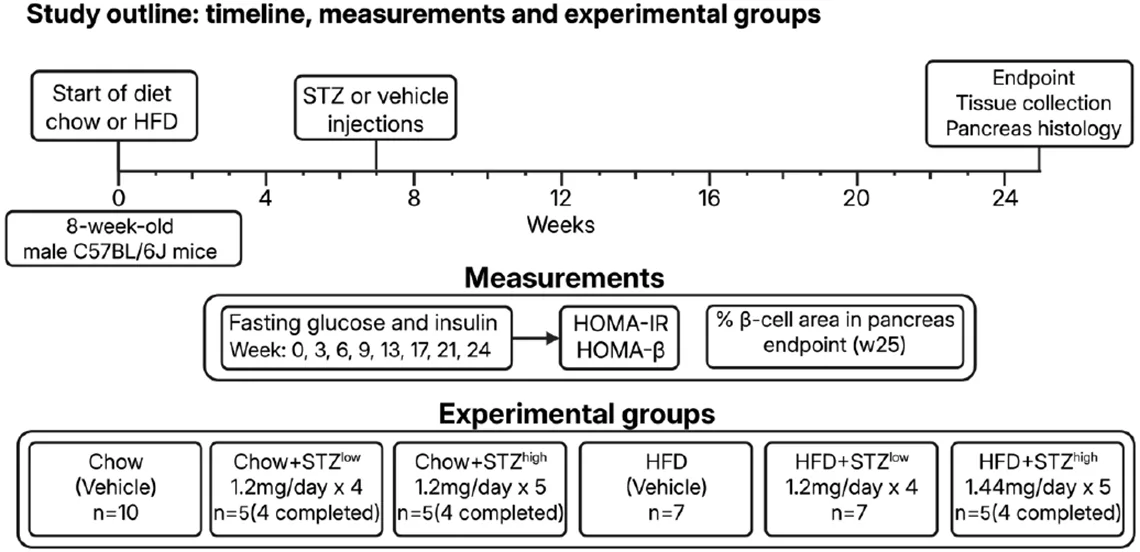
**Study outline and experimental timeline.** Eight-week-old male C57BL/6J mice were assigned to either chow or high-fat-diet (HFD) groups starting at week 0. At week 7, mice received daily intraperitoneal injections of streptozotocin (STZ) or vehicle for 4–5 consecutive days to induce β-cell dysfunction. Metabolic parameters, including fasting blood glucose and insulin, were measured at weeks 0, 3, 6, 9, 13, 17, 21 and 24, and these parameters were used to calculate indices of insulin resistance and β-cell function by the homeostatic model assessment (HOMA) method (HOMA-IR and HOMA-β, respectively). Body weight was recorded throughout the study. At terminal collection (week 25), tissues were collected, and pancreatic β-cell area was assessed histologically. Experimental groups included chow (*n*=10), chow+STZ^low^ (1.2 mg/day×4; *n*=5, *n*=4 completed), chow+STZ^high^ (1.2 mg/day×5; *n*=5, *n*=4 completed), HFD (*n*=7), HFD+STZ^low^ (1.2 mg/day×4; *n*=7) and HFD+STZ^high^ (1.44 mg/day×5; *n*=5, *n*=4 completed).

**Fig. 2.**
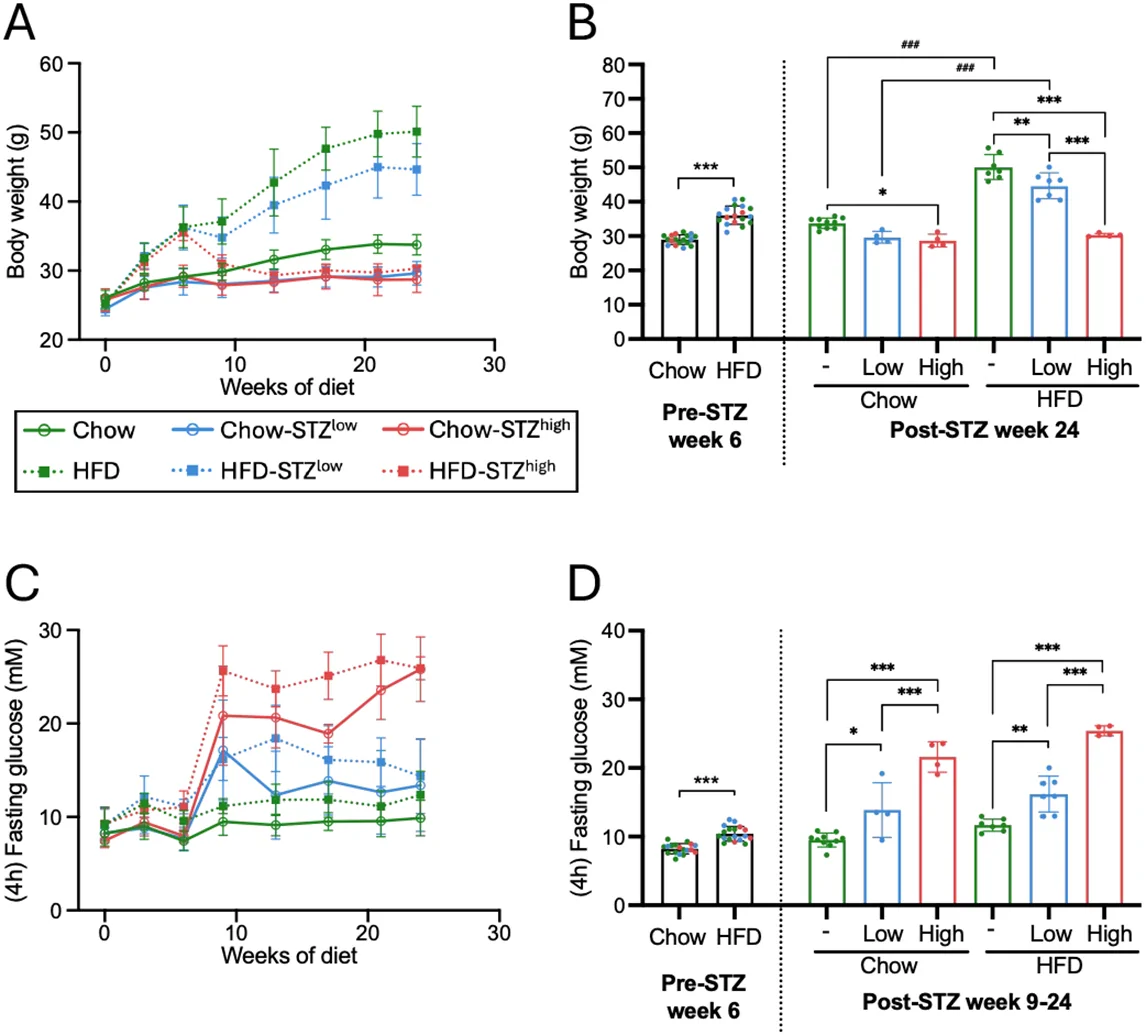
**Fixed-dose anomer-equilibrated STZ combined with HFD induces stable hyperglycaemia while preserving weight gain in HFD-STZ^low^ mice.** (A,C) Body weight (A) and fasting blood glucose (C) were measured in chow- and HFD-fed mice receiving vehicle, STZ^low^ or STZ^high^ every 3–4 weeks over 24 weeks. (B) Body weight at week 6 (pre-STZ) and week 24 (post-STZ), with the dotted line separating pre- and post-STZ data. (D) Fasting blood glucose at week 6 (pre-STZ) and the mean value from weeks 9–24 (post-STZ), with the dotted line separating pre- and post-STZ data. Pre-STZ differences between chow and HFD were analysed by unpaired two-tailed Student's *t*-test. Post-STZ differences were analysed by two-way ANOVA with diet and STZ treatment as factors, followed by Sidak's multiple-comparisons test. For post-STZ comparisons, asterisks indicate significant differences between vehicle, STZ^low^ and STZ^high^ within the same diet group, whereas hashtags indicate significant differences between chow and HFD within the same STZ/vehicle treatment condition. *P<0.05, **P<0.01, ***P<0.001; ^###^*P*<0.001. Data are shown as mean±s.d.

Fasting blood glucose levels were measured every 3–4 weeks. At week 6 (pre-STZ), HFD-fed mice exhibited a significant, mild elevation of glucose levels compared to chow-fed controls (*P*<0.001, unpaired two-tailed Student's *t*-test), and this diet effect remained stable throughout the study (Fig. 2C,D). Post-STZ (weeks 9–24), two-way ANOVA revealed significant effects of diet and STZ dose on fasting blood glucose (*P*<0.001), but no interaction (Fig. 2D). Sidak's post hoc confirmed hyperglycaemia in all STZ-treated groups relative to chow controls, with STZ^high^ causing greater elevations than STZ^low^ (Fig. 2D). To evaluate whether a fixed-dose STZ strategy influenced blood glucose differently from conventional weight-adjusted dosing, the relationship between pre-STZ body weight and post-STZ fasting blood glucose was examined in chow-STZ^low^ and HFD-STZ^low^ groups. Despite substantial variation in pre-STZ body weight (26.5–40.7 g), simple linear regression showed no association between body weight and post-STZ fasting blood glucose with fixed-dose STZ (slope=0.27, 95% c.i. −0.19 to 0.73, *R*^2^=0.16, *P*=0.21) (Fig. S1C). The positive, although non-significant, slope highlights a potential concern that weight-adjusted dosing could result in overdosing heavier mice, because these mice would receive a larger absolute STZ dose under a weight-adjusted scheme.

### HFD induces hyperinsulinemia whereas low-dose STZ normalizes insulin

In C57BL/6J mice, HFD induces proliferation of β-cells and hyperinsulinemia to compensate for insulin resistance, whereas STZ treatment causes β-cell degeneration and hypoinsulinaemia (Mosser et al., 2015). We therefore monitored fasting insulin throughout the study to assess the effects of diet and STZ dose on insulin levels. HFD-fed mice showed a significant increase in fasting insulin levels compared to those of chow-fed mice at week 6 (pre-STZ) (*P*<0.01, unpaired two-tailed Student's *t*-test) (Fig. 3A,B). Post-STZ (weeks 9–24), there was a significant main effect of diet and STZ (*P*<0.001) but no interaction (Fig. 3B). Post hoc analysis showed that the HFD group had significantly higher insulin levels than all other groups and that STZ reduced insulin levels in a dose-dependent manner (Fig. 3B). Insulin levels in HFD-STZ^low^ mice remained comparable to those in chow controls and were significantly lower than those in the HFD group (Fig. 3B). This partial reduction in insulin levels in HFD-STZ^low^ mice was sufficient to induce hyperglycaemia without causing weight loss, indicating an optimal balance between insulin levels and obesity for a sustained T2D-like phenotype.

**Fig. 3.**
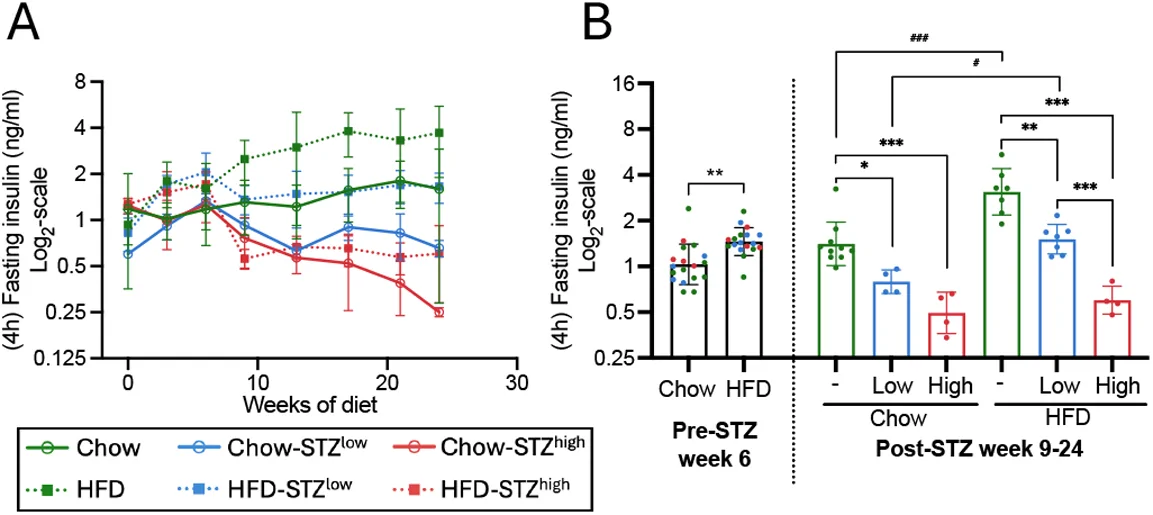
**Fixed-dose anomer-equilibrated STZ reduces fasting insulin levels in a dose-dependent manner in chow- and HFD-fed mice.** (A) Fasting serum insulin was measured in chow- and HFD-fed mice receiving vehicle, STZ^low^ or STZ^high^ every 3–4 weeks over 24 weeks. (B) Fasting insulin at week 6 (pre-STZ) and the mean value from weeks 9–24 (post-STZ), with the dotted line separating pre- and post-STZ data. Pre-STZ differences between chow and HFD were analysed by unpaired two-tailed Student's *t*-test. Post-STZ differences were analysed by two-way ANOVA with diet and STZ treatment as factors, followed by Sidak's multiple-comparisons test. For post-STZ comparisons, asterisks indicate significant differences between vehicle, STZ^low^ and STZ^high^ within the same diet group, whereas hashtags indicate significant differences between chow and HFD within the same STZ/vehicle treatment condition. *P<0.05, **P<0.01, ***P<0.001; ^#^*P*<0.05, ^###^*P*<0.001. Data were log2 transformed for statistical analysis and are shown on a log2-transformed axis. Data are shown as mean±s.d.

### HFD combined with low-dose STZ induces T2D hallmarks, with increased insulin resistance (HOMA-IR) and β-cell dysfunction (HOMA-β)

Indices of insulin resistance and β-cell function by the homeostatic model assessment (HOMA) method (HOMA-IR and HOMA-β, respectively), calculated from fasting glucose and insulin, correlate well with clamp-based measures, and were used as surrogate indices of insulin resistance and β-cell function, respectively (Wallace et al., 2004). Pre-STZ (week 6), HFD-fed mice had significantly increased insulin resistance (higher HOMA-IR) compared to chow-fed mice (*P*<0.001, unpaired two-tailed Student's *t*-test) but no significant difference in β-cell function (HOMA-β) (Fig. 4A-D). Post-STZ treatment (weeks 9–24), HOMA-IR showed significant main effects of diet and STZ dose (*P*<0.001) as well as significant diet×STZ interaction (*P*<0.05) (Fig. 4B). Post hoc analysis revealed that the HFD and HFD-STZ^low^ groups were the only groups with significantly increased HOMA-IR compared to that of chow controls (Fig. 4B). For HOMA-β, there was a significant main effect of STZ dose (*P*<0.001) but no effect of diet or interaction (Fig. 4D). Post hoc analysis showed that all STZ-treated groups exhibited marked reductions in HOMA-β compared to vehicle-treated groups, with a significantly greater reduction in STZ^high^ groups (Fig. 4D). Assessment of the relationship between HOMA-IR and HOMA-β across individual mice revealed clear clustering of metabolic phenotypes according to diet and STZ treatment (Fig. S2A). Relative to chow controls, HFD mice clustered at high HOMA-IR together with elevated HOMA-β values. HFD-STZ^low^ mice formed a distinct cluster characterized by moderately increased HOMA-IR and reduced HOMA-β. In contrast, STZ^high^ mice clustered at normal HOMA-IR and low HOMA-β values, irrespective of diet (Fig. S2A).

**Fig. 4.**
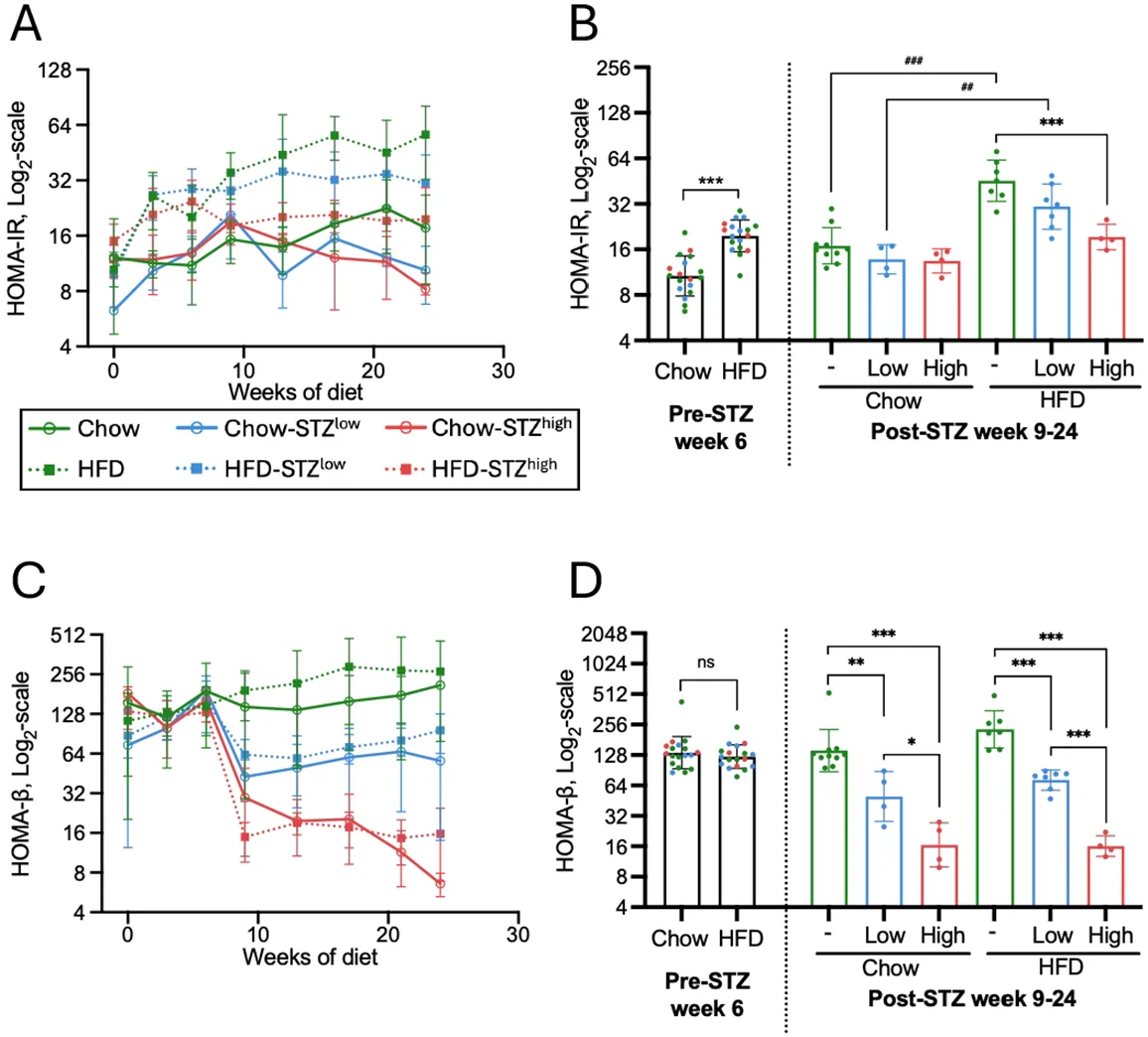
**HOMA-β and HOMA-IR reveal a robust type 2 diabetes phenotype in HFD-STZ^low^ mice.** (A,C) HOMA-IR (A) and HOMA-β (C) were measured in chow- and HFD-fed mice receiving vehicle, STZ^low^ or STZ^high^ every 3–4 weeks over 24 weeks. (B,D) HOMA-IR (B) and HOMA-β (D) at week 6 (pre-STZ), and the mean values from weeks 9–24 (post-STZ), with the dotted line separating pre- and post-STZ data. Pre-STZ differences between chow and HFD were compared by unpaired two-tailed Student's *t*-test. Post-STZ differences were analysed by two-way ANOVA with diet and STZ treatment as factors, followed by Sidak's multiple-comparisons test. For post-STZ comparisons, asterisks indicate significant differences between vehicle, STZ^low^ and STZ^high^ within the same diet group, whereas hashtags indicate significant differences between chow and HFD within the same STZ/vehicle treatment condition. ns, not significant; *P<0.05, **P<0.01, ***P<0.001; ^##^*P*<0.01, ^###^*P*<0.001. Data were log2 transformed for statistical analysis and are shown on a log2-transformed axis as mean±s.d.

### HOMA-β is a valid surrogate for β-cell loss after STZ treatment

At terminal endpoint (week 25), β-cell loss was assessed by immunohistochemistry and quantification of insulin-positive staining in pancreas. There was a significant main effect of STZ dose (*P*<0.001), but no significant effect of diet or interaction, and post hoc analysis revealed a STZ dose-dependent reduction in insulin-positive staining (Fig. 5A). The HFD-STZ^low^ group displayed significantly higher β-cell area than the HFD-STZ^high^ and chow-STZ^high^ groups, although still ∼50% lower than that in normal, vehicle-treated groups. Simple linear regression on log2-transformed data showed that HOMA-β strongly predicted the percentage of insulin-positive β-cell area (slope=0.69, 95% c.i. 0.59–0.79, *R*^2^=0.85, *P*<0.0001), indicating that HOMA-β is a good predictor of STZ-induced β-cell loss (Fig. 5B).

**Fig. 5.**
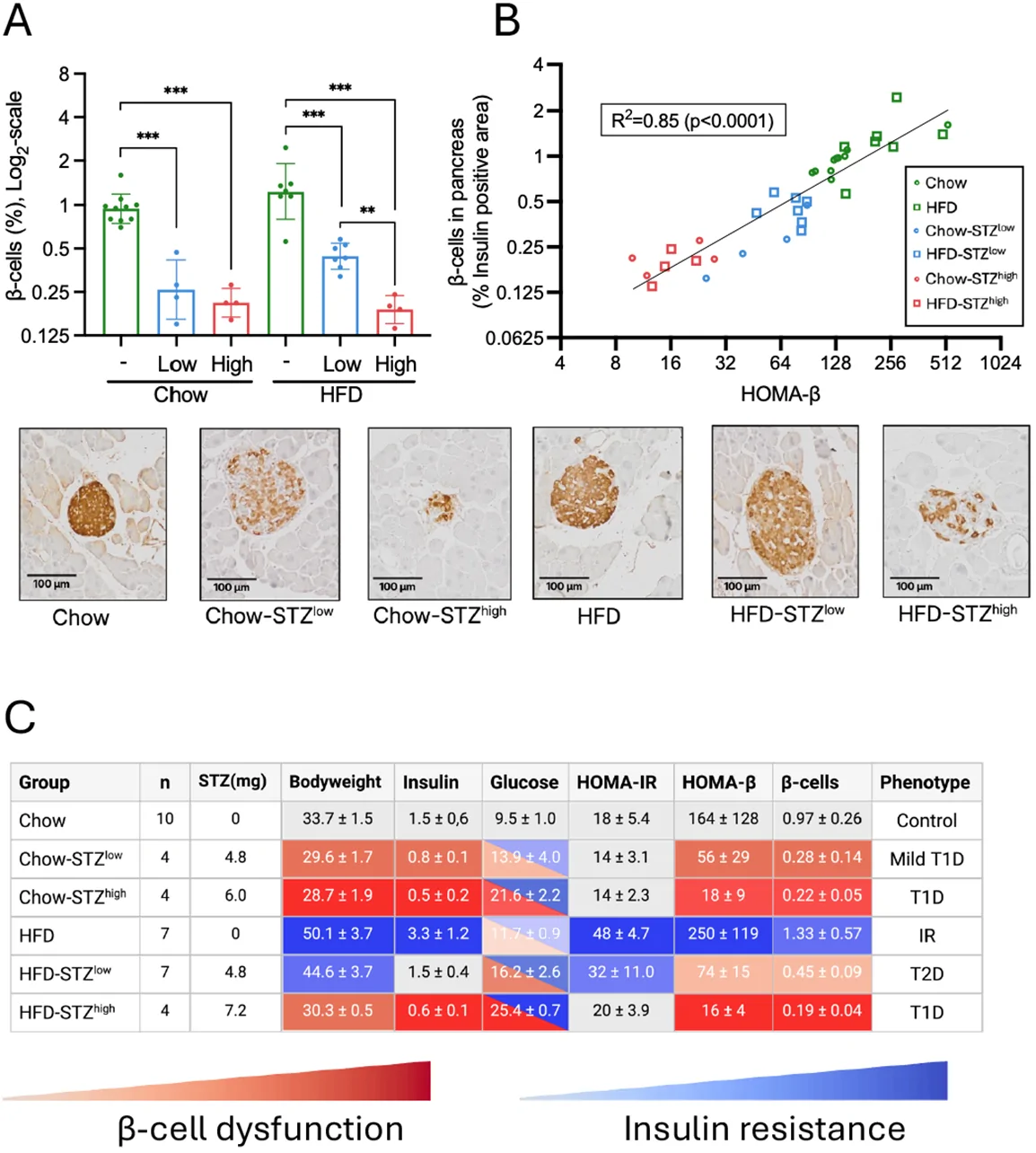
**HOMA-β predicts insulin-positive β-cell area after STZ treatment.** (A) Insulin-positive β-cell area, expressed as percentage of total pancreas area, was quantified at endpoint in chow- and HFD-fed mice receiving vehicle, STZ^low^ or STZ^high^. Data were analysed by two-way ANOVA with diet and STZ treatment as factors, followed by Sidak's multiple-comparisons test. Asterisks indicate significant differences between vehicle, STZ^low^ and STZ^high^ within the same diet group. **P<0.01, ***P<0.001. Comparisons between chow and HFD within the same STZ/vehicle treatment condition were also performed but were not significant. Data were log2 transformed for statistical analysis and are shown on a log2-transformed axis as mean±s.d. (B) Linear regression analysis of log2-transformed HOMA-β versus insulin-positive β-cell area showed a strong positive correlation (*R*^2^=0.85, slope=0.69, 95% c.i. 0.59–0.79, *P*<0.0001). Representative insulin-stained pancreatic sections are shown below. (C) Summary and overview of metabolic phenotypes across groups based on body weight, fasting insulin and glucose, HOMA-IR, HOMA-β, and β-cell quantification (insulin-positive area/total pancreas area). Values represent group mean±s.d. calculated from the average value of each mouse across weeks 9–24 post-STZ treatment, except for body weight and β-cell measurements, which were assessed at endpoint – body weight at week 24 and β-cell area at terminal collection (week 25). Red and blue colour gradients indicate increasing β-cell dysfunction and insulin resistance, respectively, while grey indicates metabolically healthy reference values (chow). Note that fasting glucose reflects both insulin resistance and β-cell dysfunction and is represented using a split red/blue gradient.

### Combination of multiple metabolic parameters across intervention groups identifies distinct models for insulin resistance, T1D and T2D

The six experimental groups allowed the combined investigation of diet- and STZ-induced effects on metabolic phenotypes (Fig. 5C). The chow vehicle group represented metabolically healthy controls and served as a reference for metabolic changes in the other groups. The HFD vehicle group displayed a phenotype characterized by insulin resistance, consistent with a prediabetic state maintained by compensatory β-cell function. The chow-STZ^low^ group exhibited mild β-cell dysfunction, consistent with early/mild T1D. Both STZ^high^ groups (chow and HFD) displayed metabolic profiles associated with severe β-cell dysfunction and hyperglycaemia, consistent with overt T1D. The HFD-STZ^low^ group was the only group exhibiting both β-cell dysfunction and insulin resistance, consistent with a T2D-like phenotype. Thus, by different combinations of diet and STZ dosing, we found distinct models for insulin resistance (prediabetes), T1D (mild/overt) and T2D in C57BL/6J mice. Additionally, the HFD-STZ^low^ group demonstrated low inter-individual variability across key T2D parameters (averaged across weeks 9–24): blood glucose (13–20 mM), HOMA-IR (18–49), HOMA-β (47–88), β-cell area (0.44–0.58%) and body weight (40.4–50.1 g) (minimum–maximum range). This finding indicates that the fixed-dose STZ strategy produced a stable T2D-like phenotype with low inter-individual variability.

### Mathematical modelling of β-cell loss after STZ treatment

We created a semi-mechanistic mathematical model to further elucidate the relationship between the STZ dosage and glucose response. The model consisted of ordinary differential equations to describe the β-cell, glucose and insulin dynamics after STZ treatment (Fig. 6A). The full model description can be found in the Supplementary Materials and Methods. The model was trained to simultaneously describe collected data at weeks 0, 6, 9 and 24 for β-cell, glucose and insulin; control, STZ^low^ and STZ^high^; for chow (Fig. 6B) and HFD (Fig. 6C). This resulted in a model behaviour representing the mean dynamics in all data. Overall, the model fits qualitatively well to all data. The best model agreement can be seen as the line in Fig. 6B,C, and the model uncertainty [best agreement+χ^2^(0.95,1)] is the shaded area. Next, the predictive capabilities of the model were validated using independent-validation fasting plasma glucose data from Gilbert et al. (2011) from mice undergoing STZ treatment with body weight-adjusted dose. The model was able to qualitatively well predict the levels of plasma glucose for the chow and HFD groups (Fig. 6D).

**Fig. 6.**
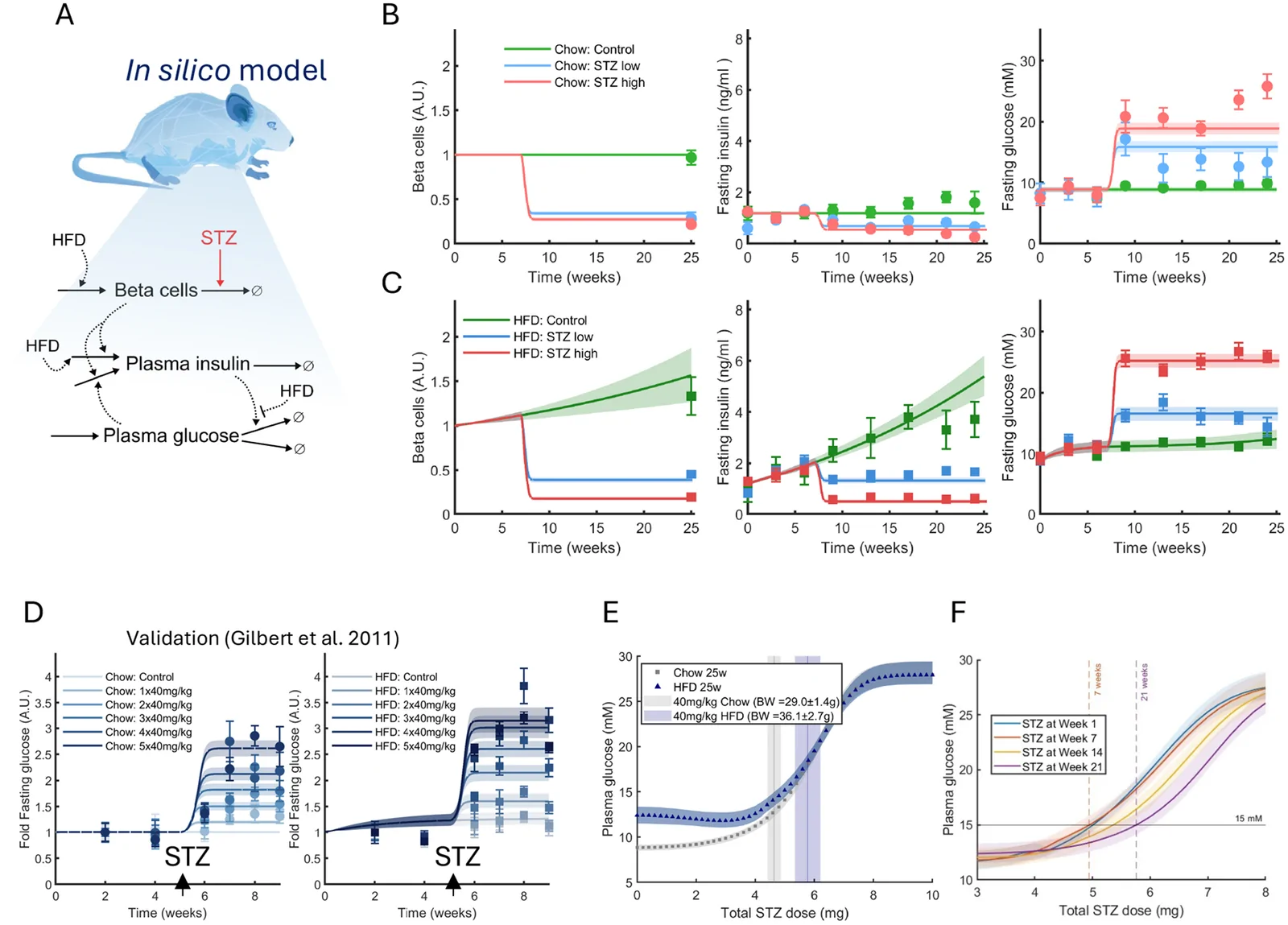
**Mathematical modelling of STZ intervention in mice.** (A) Schematic representation of the mathematical model structure. (B,C) Simultaneous agreement between model simulations and all estimation data. Here, the model simulations (lines) for vehicle (green), STZ^low^ (blue) or STZ^high^ (red) for chow (B) and HFD (C) are compared to the experimental data (error bars), for β-cells, fasting insulin and fasting plasma glucose. (D) Model validation using independent experimental data from Gilbert et al. (2011). Model predictions of fasting plasma glucose for STZ dosing schemes not included during model fitting were compared with independent experimental data. Lines represent model predictions, while points and error bars show the corresponding experimental data. (E) Model predictions of fasting plasma glucose at 25 weeks when administering different levels of total STZ doses for chow and HFD. The shapes are the endpoint of each 25 week *in silico* trial – chow is grey and HFD is blue, with the surrounding area indicating model uncertainty. The shaded vertical area is the dose interval for chow (grey) and HFD (blue) body weight-adjusted STZ doses based on the mean (±s.d.) weight in our cohort. (F) Model predictions of dose–response curve showing fasting plasma glucose endpoint after 25 weeks, but with different timing of STZ dosing scheme at week 1, 7 (our study), 14 and 21. A.U., arbitrary units; w, week.

Next, we used the validated model to predict the dose–response relationship between STZ and fasting plasma glucose. This was done by simulating several 25-week trials with a range of STZ dosages (0–10 mg total), with injections administered at week 7 over 4 consecutive days for both chow and HFD (Fig. 6E). In Fig. 6E, each dot represents the fasting plasma glucose endpoint (25 weeks) for a single simulated trial (the shaded area is the model uncertainty). The model predicts a sigmoidal relationship between the total STZ dose and endpoint plasma glucose, with maximum response of ∼25 mM at higher doses. To illustrate the potential difference in outcomes between fixed body weight-adjusted dosing, we highlight the body weight adjusted-doses based on the mean body weight (±s.d.) at week 6 (shaded boxes in Fig. 6E). Here, focusing on HFD, we can observe that the body weight-adjusted dosing (based on mean body weight) resulted in different fasting plasma glucose values (15.5–21.2 mM) compared to the endpoint using fixed dose (17.6–19.1 mM), based on data uncertainty captured by the model. Importantly, the model does not capture the full uncertainty in data (outliers); the observation is based on the mean behaviour and describes the effect of HFD without body-weight scaling. Nevertheless, this may warrant further comparison between the two dosing schemes.

Lastly, we also investigated the effect of plasma glucose on timing of the STZ intervention. To do this, we simulated four 25-week HFD interventions with the same STZ dosing schemes at different time points, at weeks 1, 7, 14 and 21 (Fig. 6F). The model predicts that a larger total STZ dose, 5.76 mg for dosing at 21 weeks compared to 4.95 mg for 7 weeks, would be needed to reach the same endpoint fasting glucose (15 mM).

## DISCUSSION

In this study, we characterized the effects of chow or HFD feeding combined with low or high STZ doses on multiple metabolic parameters over a 24-week period. We identified a robust protocol for inducing T2D in C57BL/6J mice, combining HFD feeding with anomer-equilibrated STZ administered at a fixed dose (mg) rather than body weight-adjusted (mg/kg). Historically, weight-adjusted dosing of STZ has been the standard approach for inducing diabetes in rodent models (Furman, 2021). However, studies comparing animals across differences in age, diet, sex and species consistently report more severe glycaemic responses to STZ in heavier animals (Masiello et al., 1975; Gilbert et al., 2011; Talbot et al., 2026; Furman, 2021). Although these factors (age, diet, sex and species) may each contribute to the variability in STZ sensitivity, an alternative explanation is that weight-adjusted dosing leads to relative overdosing in heavier animals, whereas fixed dosing (mg) may reduce this effect. Consistent with this, weight-adjusted dosing of hydrophilic compounds, such as STZ, is well recognized to increase the risk of overdosing in obese subjects (Lenzen, 2008; Gouju and Legeay, 2023). Thus, fixed STZ dosing, especially in obese animals, may yield more consistent glycaemic responses. In support of this, HFD-STZ^low^ mice maintained obesity and insulin resistance while developing moderate hyperglycaemia and partial β-cell loss. Longitudinal assessment showed progression from HFD-induced insulin resistance with compensatory β-cell function to overt T2D following STZ-induced β-cell loss.

Considerable heterogeneity exists in protocols that combine HFD with STZ to induce T2D in mice, with wide variation in HFD duration, STZ dose and number of injections. Similar diabetic phenotypes (∼13–19 mM fasting glucose) have been achieved using markedly different strategies, including 3–12 weeks of HFD feeding followed by either a single high-dose or multiple low-dose STZ regimens (e.g. 1×100, 3×30, 3×40, 3×45, 3×75, or 5×50 mg/kg) (Racine et al., 2024; Gilbert et al., 2011; Parilla et al., 2018; Karuna et al., 2025; Zheng et al., 2026; Deng et al., 2025). This extensive heterogeneity highlights the need for a more standardized and reproducible protocol. Based on our findings, we propose using an anomer-equilibrated fixed STZ dose (4.8 mg total, administered as 1.2 mg per day over 4 consecutive days) in mice fed an HFD for 7 weeks prior to STZ treatment. The duration of HFD feeding is critical to establish a prediabetic state, with pronounced insulin resistance before β-cell loss is induced by STZ treatment, to better mimic the progression of T2D.

HOMA-β has been used as a surrogate index for monitoring β-cell function in studies using STZ (Zhang et al., 2021, 2022). However, HOMA-β was developed and validated for human physiology, and its use in animals requires caution and has been discouraged (Wallace et al., 2004). When used, HOMA-β should be interpreted comparatively within a study, relative to a healthy control, and always in conjunction with HOMA-IR, as the index is inherently influenced by systemic insulin resistance (Wallace et al., 2004). Despite the limitations of applying this index to rodent models, we observed a strong positive correlation between HOMA-β and pancreatic β-cell mass: HOMA-β explained 85% of the variance in insulin-positive β-cell area (*R*^2^=0.85) across all diet/STZ combinations. Given that histological quantification of β-cell mass is labour intensive, HOMA-β provides a practical proxy for monitoring relative β-cell loss following STZ treatment. The β-cell loss observed in HFD-STZ^low^ mice closely mirrors that seen in humans at the time of T2D diagnosis (∼50% loss of β-cell function; Blüher et al., 2023), further adding to the suitability of this model for studying effects of T2D *in vivo* (Holman, 1998). In contrast, human β-cell loss at T1D diagnosis is typically ∼80%, although this percentage is highly variable by age of diagnosis (Chen et al., 2017). Consistent with this, STZ^high^ mice (both chow and HFD) exhibited extensive (∼80–90%) β-cell loss, indicating that HFD-STZ^high^ mice have a metabolic profile that more closely models T1D than T2D. This was also reflected by the longitudinal metabolic phenotyping, where STZ^high^ mice, irrespective of diet, displayed reduced body weight, low insulin levels and reduction in HOMA-β, but no increase in HOMA-IR, consistent with severe β-cell dysfunction but without insulin resistance. We do not suggest using HFD feeding to model T1D-like diabetes. Instead, the HFD-STZ^high^ group illustrates that excessive STZ exposure in HFD-fed mice can shift the phenotype away from T2D-like disease toward severe insulin deficiency T1D-like phenotype. When HFD/STZ is used to model T2D, preservation of obesity and insulin resistance, reflected here by maintained body weight and increased HOMA-IR, should therefore be considered alongside hyperglycaemia and β-cell dysfunction.

An important strength of this study is the extended follow-up after STZ treatment, which allowed assessment of the stability of the diabetic phenotype over time. Over weeks 9–24, the HFD-STZ^low^ group showed remarkable consistency in weekly mean glucose (14.4–18.4 mM), insulin (1.35–1.69 ng/ml), HOMA-IR (28.0–35.7) and HOMA-β (58.9–96.0) (minimum–maximum range of weekly averages), indicating a sustained T2D-like state. This is notable given that other studies have reported a high remission rate of hyperglycaemia over longer follow-up periods, and it further suggests that fixed STZ dosing combined with α/β-anomer equilibration may reduce long-term variability in STZ-based T2D models (Li et al., 2025).

Herein, we also constructed a data-driven semi-mechanistic mathematical model capable of simultaneously describing STZ interventions (low and high doses) and vehicle (control) for chow and HFD mice. The model predictions of plasma glucose dynamics were also validated using independent-validation data from Gilbert et al. (2011). There exist some previous modelling efforts of HFD and insulin resistance in mice. We have previously published a mathematical model of HFD-induced insulin resistance with which we also explored β-cell dynamics (Simonsson et al., 2023). This model had a short-term focus (2 weeks), and did not include STZ intervention or β-cell data. Thus, our new mathematical model makes a valuable contribution for simulating longer diets and also STZ interventions. The model predicted a sigmoidal dose–response relationship between STZ dose (mg) and fasting plasma glucose levels. A similar sigmoidal dose–response relationship has been observed in rats (Ghasemi and Jeddi, 2023). The model prediction highlights that body weight-adjusted dosing in HFD-fed mice may introduce high variance in fasting plasma glucose.

This study has limitations. One is that the initial high-dose STZ regimen was calculated from the average body weight within each diet group, resulting in a higher absolute STZ dose in HFD-fed mice (total 7.2 mg) than in chow-fed mice (total 6.0 mg). This may have contributed to the stronger hyperglycaemic response observed in the HFD-STZ^high^ group and should, therefore, be considered when interpreting comparisons involving this group. In addition, fixed dosing (mg) was not directly compared with conventional weight-adjusted dosing (mg/kg) in parallel experimental groups. Such a comparison would have allowed more robust conclusions regarding the relative effects of fixed versus weight-adjusted STZ dosing.

Moreover, the current mathematical model has some limitations. The model agreement does not pass a formal χ^2^-test (when including all data simultaneously) on the residuals but still fit qualitatively well to all data. Reasons for not passing a χ^2^-test is partially because we do not allow for any difference in parameter values (except for inputs) between cohorts, and we do not yet include mechanisms for age-related insulin sensitivity. Other major limitations with the current model are the lack of body weight scaling in the pharmacokinetic model, e.g. the volume of distribution, as well as body weight scaling of increased insulin demand with increasing fat mass. Moreover, the model could, in future iteration, also benefit from adding a description of alpha-cell dynamics and glucagon regulation. Thus, the model could be improved in future work by including these missing aspects, and extended with a more mechanistic description of β-cell mass and insulin resistance progression, as well as extended with mathematical models for body-weight changes (e.g. Guo and Hall, 2009), to incorporate STZ effect on fat storage capabilities.

In summary, fixed-dose, anomer-equilibrated STZ administered after HFD feeding induced a stable T2D-like phenotype with low inter-individual variability over an extended follow-up period. This strategy may be particularly useful in HFD-fed mice, in which variability in body weight can strongly influence conventional mg/kg dosing and contribute to inconsistent glycaemic outcomes.

## MATERIALS AND METHODS

### Animals and study outline

C57BL/6J mice were purchased from Taconic and maintained by in-house breeding. All animal experiments and procedures were approved by the Lund-Malmö Committee for Animal Experiment Ethics, approval number 4992/2022. Male offspring mice were housed in groups of four to five mice per cage, with *ad libitum* access to food and water. At 8 weeks of age (study week 0), mice were assigned to either an HFD (60% kcal from fat, #D12492, Research Diets) (*n*=19) or chow control diet (14% kcal from fat, #SAFE A30, SAFE Diets, France) (*n*=20). The nutritional composition of both diets is provided in Table S1. At week 7, mice received STZ (see details below) or vehicle for 4–5 consecutive days (see Fig. 1 for study outline). The experimental groups were as follows: chow vehicle (*n*=10), HFD vehicle (*n*=7), chow-STZ^low^ (*n*=5), HFD-STZ^low^ (*n*=7), chow-STZ^high^ (*n*=5) and HFD-STZ^high^ (*n*=5). The terminal endpoint was week 25, and body weight was measured throughout the study. One mouse in each of the HFD-STZ^high^ and chow-STZ^high^ groups was euthanized owing to blood glucose ≥30 mM (humane endpoint). One mouse in the chow-STZ^low^ group died during handling.

### STZ treatment

STZ (≥75% α-anomer basis, S0130-100, Sigma-Aldrich) was dissolved to a concentration of 12 mg/ml in sterile filtered (Filtropur S, 0.2 μm, Sarstedt) sodium citrate buffer (0.05 M, pH 4.5) ≥2 h prior to the first injection to ensure equilibrium (44:56) between α- and β-anomers (de la Garza-Rodea et al., 2010). The vial with STZ was kept dark at 4°C and used for subsequent days (4–5 days). At week 7, STZ was administered by intraperitoneal injection to non-fasted mice (Chaudhry et al., 2013), while vehicle mice received sodium citrate buffer.

Two STZ protocols were used, referred to as STZ^high^ and STZ^low^. STZ dosing was selected based on the protocol from Gilbert et al. (2011) and two pilot experiments using 40 mg/kg STZ for three or five consecutive injections. Based on these pilot experiments, five consecutive injections were selected for the STZ^high^ protocol. For the STZ^high^ groups, the daily STZ dose was calculated as 40 mg/kg using the average body weight within each diet group. At the time of injection, chow-fed mice weighed, on average, 30 g and therefore received 1.2 mg/day for 5 days, corresponding to 6.0 mg total STZ. HFD-fed mice weighed, on average, 36 g and therefore received 1.44 mg/day for 5 days, corresponding to 7.2 mg total STZ. Thus, within the STZ^high^ protocol, the higher absolute dose in HFD-fed mice resulted from their higher average body weight.

Because HFD-fed mice showed a marked increase in blood glucose after this regimen, the dosage was adjusted for the STZ^low^ groups by using a lower total fixed absolute dose. This dose was based on 40 mg/kg using the average body weight of chow-fed mice (30 g), corresponding to 1.2 mg/day, and was administered for 4 instead of 5 consecutive days. Thus, both chow-fed and HFD-fed mice in the STZ^low^ groups received 1.2 mg/day STZ for 4 days, corresponding to 4.8 mg total STZ.

Mice receiving STZ were supplemented with 10% sucrose in their drinking water for 7 days post injection to prevent hypoglycaemia.

### Tissue collection and assessment of pancreatic β-cell area

At experimental endpoint, mice were euthanized by intraperitoneal injection of pentobarbital (100–200 mg/kg), followed by transcardial perfusion with saline, followed by 4% paraformaldehyde (PFA). Epididymal and inguinal fat depots were dissected and weighed. Pancreas was dissected, post-fixed in 4% PFA overnight at 4°C, dehydrated in 70% ethanol and paraffin embedded. Sections were cut using a Leica RM2265 microtome. Blocks were trimmed until the complete pancreas outline was visible (0 μm), and four to six sections of 4 μm thickness were collected at +0, +400 and +800 μm intervals. Sections were transferred to slides (Superfrost Plus) and then deparaffinized in xylene, rehydrated through graded ethanol and subjected to antigen retrieval (10 mM sodium citrate, pH 6.0, 40 min, 95°C), followed by quenching of endogenous peroxidase activity (3% H_2_O_2_, 10 min). Samples were blocked in 5% goat serum in 0.25% PBST (PBS containing 0.25% Triton X-100) and incubated overnight at 4°C with a rabbit anti-insulin primary antibody (1:1000, Cell Signaling Technology, 3014T) in the same blocking buffer. Secondary staining was performed with biotinylated goat anti-rabbit [1:200, Vector Laboratories, BA-1000, 1 h, room temperature (RT)], followed by ABC-HRP (Avidin/Biotin complex, Vector Laboratories, PK-6100, 30 min, RT) and DAB chromogen (3,3'-diaminobenzidine, Vector Laboratories, SK-4100, 5 min, RT) according to the manufacturer's instructions. Slides were counterstained with Haematoxylin, dehydrated and coverslipped using Pertex (Histolab, Sweden). Whole-slide images at 20× magnification from pancreas sections collected at +400 μm and +800 μm were acquired using an Olympus BX61 with VS120-L100 automated stage (Olympus, Japan). Images were analysed in a masked manner, using QuPath (Bankhead et al., 2017). The percentage of β-cell area was quantified by measuring insulin immunopositive area over total pancreatic area via Haematoxylin and DAB channel thresholding; unspecific background staining outside the islets was manually excluded.

### Blood sampling and calculation of HOMA-IR and HOMA-β

Fasting blood glucose and serum insulin levels were measured in mice following a 4-h fast (07:00–11:00). Blood was collected from the lateral saphenous vein, and glucose levels were determined using a handheld glucometer (Contour XT, Bayer). Approximately 20–30 μl of blood was collected into serum/clotting activator tubes (Microvette^®^ CB 300, Sarstedt) and allowed to coagulate (15 min, RT). Samples were then centrifuged (1500 ***g***, 10 min, 4°C), and the resulting serum was collected and stored at −80°C. Serum insulin concentrations were quantified using an enzyme-linked immunosorbent assay (ELISA) kit (Crystal Chem, 90080) according to the manufacturer's instructions. Indices of insulin resistance (IR) and β-cell function (β) were calculated using the homeostatic model assessment (HOMA) method, as previously described (Matthews et al., 1985). HOMA-IR was calculated as insulin (μU/ml)×glucose (mM)/22.5, and HOMA-β was calculated as (20×insulin (μU/ml))/[glucose (mM)−3.5].

### Mathematical modelling

The mathematical model was constructed using ordinary differential equations with all formal analysis performed in MATLAB 2023b (MathWorks Inc., Natick, MA, USA) using the systems biology toolbox (Schmidt and Jirstrand, 2006). The full list of assumptions and model equations can be found in the Supplementary Materials and Methods. The model parameter estimation was performed using the MEIGO toolbox in MATLAB (Egea et al., 2014).

### Data and statistical analysis

GraphPad Prism v.10.6.1 (GraphPad Software) was used for statistical analyses. Pre-STZ baseline variables at week 6 were compared between chow and HFD groups using unpaired two-tailed Student's *t*-test. A two-way ANOVA was performed on data post STZ (average across weeks 9–24), with factors diet (chow, HFD) and STZ dose (vehicle, low, high), followed by Sidak's post hoc multiple comparisons test. Assumptions of normal distribution and homoscedasticity of residuals were evaluated using the Shapiro–Wilk test, Q–Q plots and residuals-versus-predicted plots. Variables that did not meet these assumptions were log2 transformed and re-analysed. Fasting insulin, HOMA-IR, HOMA-β and percentage of β-cells required log2 transformation and were, therefore, analysed using two-way ANOVA on log2-transformed data.

### Use of AI tools

ChatGPT was used to support manuscript preparation by assisting with the initial drafting process and improving text for clarity, flow and readability.

## Supplementary Material

10.1242/dmm.053071_sup1 Supplementary information

Dataset 1. Raw data

## References

[DMM053071C1] Bankhead, P., Loughrey, M. B., Fernandez, J. A., Dombrowski, Y., McArt, D. G., Dunne, P. D., McQuaid, S., Gray, R. T., Murray, L. J., Coleman, H. G. et al. (2017). QuPath: open source software for digital pathology image analysis. *Sci. Rep.* 7, 16878. 10.1038/s41598-017-17204-5 PMC5715110 29203879

[DMM053071C40] Blüher, M., Malhotra, A. and Bader, G. (2023). Beta-cell function in treatment-naïve patients with type 2 diabetes mellitus: Analyses of baseline data from 15 clinical trials. *Diabetes Obes. Metab.* 25, 1403-1407. 10.1111/dom.14969 36594703

[DMM053071C2] Chaudhry, Z. Z., Morris, D. L., Moss, D. R., Sims, E. K., Chiong, Y., Kono, T. and Evans-Molina, C. (2013). Streptozotocin is equally diabetogenic whether administered to fed or fasted mice. *Lab. Anim.* 47, 257-265. 10.1177/0023677213489548 PMC3773005 23760565

[DMM053071C3] Chen, C., Cohrs, C. M., Stertmann, J., Bozsak, R. and Speier, S. (2017). Human beta cell mass and function in diabetes: recent advances in knowledge and technologies to understand disease pathogenesis. *Mol. Metab.* 6, 943-957. 10.1016/j.molmet.2017.06.019 PMC5605733 28951820

[DMM053071C4] de la Garza-Rodea, A. S., Knaan-Shanzer, S., den Hartigh, J. D., Verhaegen, A. P. and van Bekkum, D. W. (2010). Anomer-equilibrated streptozotocin solution for the induction of experimental diabetes in mice (Mus musculus). *J. Am. Assoc. Lab. Anim. Sci.* 49, 40-44. PMC2824966 20122315

[DMM053071C5] Deng, L., Yan, F., Feng, C. and Yang, Q. (2025). Multi-omics analysis and nephroseq database of genes related to kidney function in diabetic nephropathy patients to predict potential target drugs. *Diabetes Metab. Syndr. Obes.* 18, 3781-3794. 10.2147/DMSO.S526696 PMC12513384 41078586

[DMM053071C6] Egea, J. A., Henriques, D., Cokelaer, T., Villaverde, A. F., MacNamara, A., Danciu, D. P., Banga, J. R. and Saez-Rodriguez, J. (2014). MEIGO: an open-source software suite based on metaheuristics for global optimization in systems biology and bioinformatics. *BMC Bioinform.* 15, 136. 10.1186/1471-2105-15-136 PMC4025564 24885957

[DMM053071C7] Emerging Risk Factors Collaboration, Sarwar, N., Gao, P., Seshasai, S. R., Gobin, R., Kaptoge, S., Di Angelantonio, E., Ingelsson, E., Lawlor, D. A., Selvin, E., Stampfer, M., et al. (2010). Diabetes mellitus, fasting blood glucose concentration, and risk of vascular disease: a collaborative meta-analysis of 102 prospective studies. *Lancet* 375, 2215-2222. 10.1016/S0140-6736(10)60484-9 PMC2904878 20609967

[DMM053071C8] Furman, B. L. (2021). Streptozotocin-induced diabetic models in mice and rats. *Curr. Protoc.* 1, e78. 10.1002/cpz1.78 33905609

[DMM053071C9] GBD Diabetes Collaborators (2023). Global, regional, and national burden of diabetes from 1990 to 2021, with projections of prevalence to 2050: a systematic analysis for the Global Burden of Disease Study 2021. *Lancet* 402, 203-234. 10.1016/S0140-6736(23)01301-6 PMC10364581 37356446

[DMM053071C10] GBD Diseases Injuries Collaborators (2020). Global burden of 369 diseases and injuries in 204 countries and territories, 1990-2019: a systematic analysis for the Global Burden of Disease Study 2019. *Lancet* 396, 1204-1222. 10.1016/S0140-6736(20)30925-9 PMC7567026 33069326

[DMM053071C11] Ghasemi, A. and Jeddi, S. (2023). Streptozotocin as a tool for induction of rat models of diabetes: a practical guide. *EXCLI J.* 22, 274-294. 10.17179/excli2022-5720 PMC10043433 36998708

[DMM053071C12] Gilbert, E. R., Fu, Z. and Liu, D. (2011). Development of a nongenetic mouse model of type 2 diabetes. *Exp. Diabetes Res.* 2011, 416254. 10.1155/2011/416254 PMC3226533 22164157

[DMM053071C13] Gouju, J. and Legeay, S. (2023). Pharmacokinetics of obese adults: not only an increase in weight. *Biomed. Pharmacother.* 166, 115281. 10.1016/j.biopha.2023.115281 37573660

[DMM053071C14] Goyal, S. N., Reddy, N. M., Patil, K. R., Nakhate, K. T., Ojha, S., Patil, C. R. and Agrawal, Y. O. (2016). Challenges and issues with streptozotocin-induced diabetes - A clinically relevant animal model to understand the diabetes pathogenesis and evaluate therapeutics. *Chem. Biol. Interact.* 244, 49-63. 10.1016/j.cbi.2015.11.032 26656244

[DMM053071C15] Guo, J. and Hall, K. D. (2009). Estimating the continuous-time dynamics of energy and fat metabolism in mice. *PLoS Comput. Biol.* 5, e1000511. 10.1371/journal.pcbi.1000511 PMC2731929 19763167

[DMM053071C16] Holman, R. R. (1998). Assessing the potential for alpha-glucosidase inhibitors in prediabetic states. *Diabetes Res. Clin. Pract.* 40 Suppl, S21-S25. 10.1016/S0168-8227(98)00038-2 9740498

[DMM053071C17] Kamli-Salino, S. E. J., Brown, P. A. J., Haschler, T. N., Liang, L., Feliers, D., Wilson, H. M. and Delibegovic, M. (2023). Induction of experimental diabetes and diabetic nephropathy using anomer-equilibrated streptozotocin in male C57Bl/6J mice. *Biochem. Biophys. Res. Commun.* 650, 109-116. 10.1016/j.bbrc.2023.01.089 36774688

[DMM053071C18] Karuna, N., Kerrigan, L., Edgar, K., Cappa, O., Simpson, D., Tonry, C., Grieve, D. J. and Watson, C. J. (2025). Deep phenotyping of a modified diabetic cardiomyopathy mouse model which reflects clinical disease progression. *Diabetol. Metab. Syndr.* 17, 334. 10.1186/s13098-025-01913-3 PMC12351925 40814000

[DMM053071C19] Kleinert, M., Clemmensen, C., Hofmann, S. M., Moore, M. C., Renner, S., Woods, S. C., Huypens, P., Beckers, J., de Angelis, M. H., Schurmann, A. et al. (2018). Animal models of obesity and diabetes mellitus. *Nat. Rev. Endocrinol.* 14, 140-162. 10.1038/nrendo.2017.161 29348476

[DMM053071C20] Lenzen, S. (2008). The mechanisms of alloxan- and streptozotocin-induced diabetes. *Diabetologia* 51, 216-226. 10.1007/s00125-007-0886-7 18087688

[DMM053071C21] Li, H., Qin, F., Zheng, S., Wu, J., Lin, S., Gao, X. and Liu, Y. (2025). Construction and evaluation of STZ-induced diabetes and diabetic kidney disease models in C57BL/6J mice. *Front. Endocrinol. (Lausanne)* 16, 1711035. 10.3389/fendo.2025.1711035 PMC12753433 41480368

[DMM053071C22] Masiello, P., De Paoli, A. and Bergamini, E. (1975). Age-dependent changes in the sensitivity of the rat to a diabetogenic agent (streptozotocin). *Endocrinology* 96, 787-789. 10.1210/endo-96-3-787 123196

[DMM053071C23] Matthews, D. R., Hosker, J. P., Rudenski, A. S., Naylor, B. A., Treacher, D. F. and Turner, R. C. (1985). Homeostasis model assessment: insulin resistance and beta-cell function from fasting plasma glucose and insulin concentrations in man. *Diabetologia* 28, 412-419. 10.1007/BF00280883 3899825

[DMM053071C24] Mosser, R. E., Maulis, M. F., Moulle, V. S., Dunn, J. C., Carboneau, B. A., Arasi, K., Pappan, K., Poitout, V. and Gannon, M. (2015). High-fat diet-induced beta-cell proliferation occurs prior to insulin resistance in C57Bl/6J male mice. *Am. J. Physiol. Endocrinol. Metab.* 308, E573-E582. 10.1152/ajpendo.00460.2014 PMC4385873 25628421

[DMM053071C25] NCD Risk Factor Collaboration (2024). Worldwide trends in diabetes prevalence and treatment from 1990 to 2022: a pooled analysis of 1108 population-representative studies with 141 million participants. *Lancet* 404, 2077-2093. 10.1016/S0140-6736(24)02317-1 PMC7616842 39549716

[DMM053071C26] Parilla, J. H., Willard, J. R., Barrow, B. M. and Zraika, S. (2018). A mouse model of Beta-cell dysfunction as seen in human type 2 diabetes. *J. Diabetes Res.* 2018, 6106051. 10.1155/2018/6106051 PMC5952555 29854823

[DMM053071C27] Pearson, E. R. (2019). Type 2 diabetes: a multifaceted disease. *Diabetologia* 62, 1107-1112. 10.1007/s00125-019-4909-y PMC6560016 31161345

[DMM053071C28] Povoski, S. P., McCullough, P. J., Zhou, W. and Bell, R. H. Jr. (1993). Induction of diabetes mellitus in Syrian golden hamsters using stored equilibrium solutions of streptozotocin. *Lab. Anim. Sci.* 43, 310-314. 8231086

[DMM053071C29] Prasad, R. B. and Groop, L. (2015). Genetics of type 2 diabetes-pitfalls and possibilities. *Genes (Basel)* 6, 87-123. 10.3390/genes6010087 PMC4377835 25774817

[DMM053071C30] Racine, K. C., Iglesias-Carres, L., Herring, J. A., Wieland, K. L., Ellsworth, P. N., Tessem, J. S., Ferruzzi, M. G., Kay, C. D. and Neilson, A. P. (2024). The high-fat diet and low-dose streptozotocin type-2 diabetes model induces hyperinsulinemia and insulin resistance in male but not female C57BL/6J mice. *Nutr. Res.* 131, 135-146. 10.1016/j.nutres.2024.09.008 39389000

[DMM053071C31] Schmidt, H. and Jirstrand, M. (2006). Systems Biology Toolbox for MATLAB: a computational platform for research in systems biology. *Bioinformatics* 22, 514-515. 10.1093/bioinformatics/bti799 16317076

[DMM053071C32] Simonsson, C., Lövfors, W., Bergqvist, N., Nyman, E., Gennemark, P., Stenkula, K. G. and Cedersund, G. (2023). A multi-scale in silico mouse model for diet-induced insulin resistance. *Biochem. Eng. J.* 191, 108798. 10.1016/j.bej.2022.108798

[DMM053071C33] Singh, R., Gholipourmalekabadi, M. and Shafikhani, S. H. (2024). Animal models for type 1 and type 2 diabetes: advantages and limitations. *Front. Endocrinol. (Lausanne)* 15, 1359685. 10.3389/fendo.2024.1359685 PMC10912558 38444587

[DMM053071C34] Talbot, S. R., Heider, M., Wirth, M., Jorns, A. and Naujok, O. (2026). Comparative analysis of dose-response variability and severity in STZ-induced diabetes: female vs. male NSG mice. *Sci. Rep.* 16, 8257. 10.1038/s41598-026-42408-z PMC12963432 41786859

[DMM053071C35] Wallace, T. M., Levy, J. C. and Matthews, D. R. (2004). Use and abuse of HOMA modeling. *Diabetes Care* 27, 1487-1495. 10.2337/diacare.27.6.1487 15161807

[DMM053071C36] Wang, Z. and Gleichmann, H. (1998). GLUT2 in pancreatic islets: crucial target molecule in diabetes induced with multiple low doses of streptozotocin in mice. *Diabetes* 47, 50-56. 10.2337/diab.47.1.50 9421374

[DMM053071C37] Zhang, B., Yin, R., Lang, J., Yang, L., Zhao, D. and Ma, Y. (2021). PM(2.5) promotes beta cell damage by increasing inflammatory factors in mice with streptozotocin. *Exp. Ther. Med.* 22, 832. 10.3892/etm.2021.10264 PMC8200811 34149878

[DMM053071C38] Zhang, C., Zhang, D., Wang, H., Lin, Q., Li, M., Yuan, J., Gao, G. and Dong, J. (2022). Hyperbaric oxygen treatment improves pancreatic beta-cell function and hepatic gluconeogenesis in STZ-induced type-2 diabetes mellitus model mice. *Mol. Med. Rep.* 25, 90. 10.3892/mmr.2022.12606 PMC8809048 35039874

[DMM053071C39] Zheng, J., Wang, X., Guo, Y. and Feng, L. (2026). A novel mechanism of chlorogenic acid against type 2 diabetes-induced diabetic retinopathy: suppressing ferroptosis via NRF2/xCT/GPX4 and STAT3 signaling. *Naunyn Schmiedebergs Arch. Pharmacol.* 399, 11721-11741. 10.1007/s00210-026-05106-1 41729256

