# Supplementary material for: Fixed-dose streptozotocin combined with high-fat diet induces a stable type 2 diabetes-like phenotype in C57BL/6J mice
Source: Dis Model Mech. 2026 Sep 17;19(9):dmm053071. doi: 10.1242/dmm.053071 (PMC13617974; doi:10.1242/dmm.053071)
Supplement: Supplementary file 1 [file dmm-19-053071-s1.pdf]

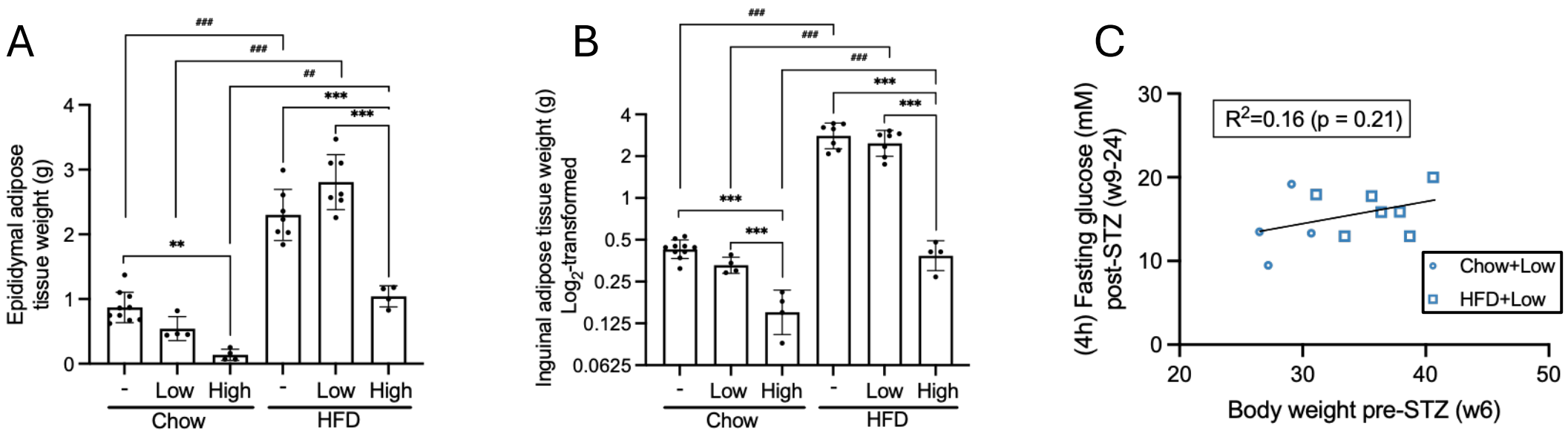

Fig. S1. Adipose tissue weights and relationship between pre-STZ body weight and glycaemic response in the chow-STZ^{low} and HFD-STZ^{low} groups.

(A) Epididymal adipose tissue weight and (B) inguinal adipose tissue weight were measured at endpoint in chow- and HFD-fed mice receiving vehicle, STZ^{low}, or STZ^{high}. Inguinal adipose tissue weights are shown as log2-transformed values. Data were analysed by two-way ANOVA with diet and STZ treatment as factors, followed by Sidak's multiple-comparisons test, asterisks indicate significant differences between vehicle, STZ^{low}, and STZ^{high} within the same diet group, whereas hashtags indicate significant differences between chow and HFD within the same STZ/vehicle treatment condition. *p < 0.05, **p < 0.01, ***p < 0.001; #p < 0.05, ##p < 0.01, ###p < 0.001. Data are displayed as mean ± SD. (C) Relationship between pre-STZ body weight at week 6 and post-STZ fasting glucose (mean weeks 9–24) in mice receiving a fixed dose of STZ. The analysis was performed to test whether mice with higher body weight at the time of injection showed a weaker glycaemic response to the same STZ dose. Linear regression showed no significant association between pre-STZ body weight and post-STZ fasting glucose (R^2=0.16, slope= 0.27, 95% CI [-0.19 to 0.73], p=0.21).

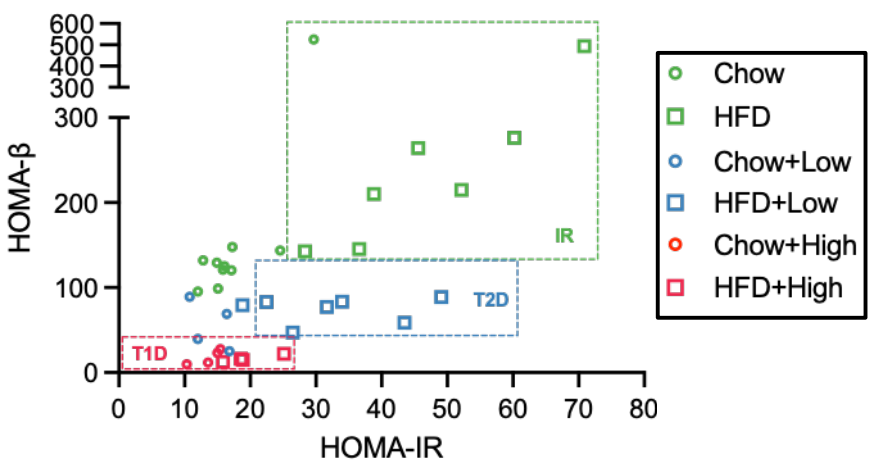

Fig. S2. Relationship between HOMA-IR and HOMA-β (mean weeks 9-24) in chow- and HFD-fed mice receiving vehicle, STZ^{low}, or STZ^{high}. Outlined boxes indicate clustering of metabolic phenotypes: insulin resistance (high HOMA-IR, high HOMA-β) in HFD vehicle, T2D (high HOMA-IR, moderately reduced HOMA-β) in HFD-STZ^{low}, and T1D (normal HOMA-IR, low HOMA-β) for chow-STZ^{high} and HFD-STZ^{high}. Chow vehicle serves as a healthy control reference.

Table S1. Composition of chow and HFD characteristics and composition

Characteristic	Chow diet	High-fat diet (HFD)
Product	SAFE A30	Research Diets D12492
Diet type	Grain-based complete rodent diet	Purified high-fat diet
Energy density	3.39 kcal/g	5.2 kcal/g
**By weight**		
Protein, % by weight	22.0	26
Carbohydrate/NFE, % by weight	50.8	26
Fat, % by weight	5.3	35
**By energy %**		
Energy from protein, %	26.0	20
Energy from carbohydrate/NFE, %	60.0	20
Energy from fat, %	14.1	60

HFD: *#D12492 from Research diets*: Casein, Lactic, 30 Mesh, Cystine, L Lodex 10 Sucrose, Fine Granulated Solka Floc, FCC200 Lard Soybean Oil, USP S10026B (mineral) Choline Bitartrate V10001C (vitamin) Dye, Blue FD&C #1, Alum. Lake 35-42%.
https://researchdiets.com/en/formulas/d12492

Chow: *#SAFE®A30 from Safe-lab:* Wheat, soybean meal, maize, wheat bran, barley, extruded soybeans, maize gluten, wheat germ, sunflower seed, inactivated brewer's yeast, calcium carbonate, pre-mixture of vitamins, dicalcium phosphate, pre mixture of minerals, L-lysine, sodium chloride.
https://safe-lab.com/safe-wAssets/docs/product-data-sheets/diets/tds_safe_a30_en_2407_publish.pdf

Dataset 1. Raw data

Available for download at
https://journals.biologists.com/dmm/article-lookup/doi/10.1242/dmm.053071#supplementary-data

Disease Models & Mechanisms • Supplementary information

Supplementary Materials and Methods

Model description

This section will go through the mathematical model equation and all underlaying assumptions. The model analysis was done using MATLAB 2023b and the Systems Biology Toolbox (1). The model was developed using iterative hypothesis testing, with a multiride of candidate model structure being evaluated until one was found that could describe data sufficiently well.

The model was developed for the purpose of describing data for the levels of β-cells (A.U.), fasting plasma glucose (mM) and fasting plasma insulin (ng/mL) for controls, and STZ interventions in chow and HFD mice. To describe the Streptozotocin (STZ) intervention we implemented a one-compartment first-order kinetic pharmacokinetic model for describing the level of STZ in plasma (parameters: $k_{clc_{stz}}$, V_{stz} and $k_{a_{stz}}$). The parameter starting point where chosen based on the assumption of rapid plasma clearance of STZ. The state representing STZ in plasma was then allowed to permanently lower the levels β-cells in the model. The β-cells were represented by the following equation:

$$\frac{d}{dt}\,\beta = \mathrm{IR}_\beta - \mathrm{damage}_\beta,\ \ \beta(0) = 1 \quad \textbf{(Eq.1)}$$

Where IR_β is a variable describing the increase of β cells during HFD, written as:

$$\mathrm{IR}_\beta = \begin{cases} ff > 0.5, & k_{hfd} \cdot \max(0, \beta - \mathrm{th}) \\ & else\ 0 \end{cases} \quad \textbf{(Eq.2)}$$

where k_{hfd} (d^{-1}) governs the increase in beta cells due to HFD, and *th* (a.u.) is the threshold for when enough beta cells are lost due to STZ to not have any diet-induced increase. The *damage* term was a hill equation:

$$\mathrm{damage}_\beta = \frac{\beta \cdot C^2 \cdot k_{stz}}{km_{stz}^2 + C^2} \quad \textbf{(Eq .3)}$$

where C is plasma concentration of STZ, the maximal damage rate· k_{stz} and half-saturation constant km_{stz}. To keep the model uncertainty lower we first optimized the STZ and the effect on β cells for all data and then kept the parameters governing STZ constant. To describe the effect on glucose-insulin interplay the model also included states representing fasting insulin and glucose. These state were loosely based on the glucose and insulin equation in the Topp et al. model (2). The fasting insulin was described as:

$$\frac{d}{dt}\,\mathrm{I} = \beta_\mathrm{f}\left(\sigma_{Ibasal} \cdot IR_I + \frac{\sigma_I \cdot G^2}{\alpha_I + G^2}\right) - k_{clc} \cdot \mathrm{I}\,, \mathrm{I}(0) = 10 \quad \textbf{(Eq .4)}$$

$$\beta_\mathrm{f} = \frac{\beta^2}{km_\beta + \beta^2} \quad \textbf{(Eq .5)}$$

$$\frac{d}{dt} IR_I = \begin{cases} ff > 0.5, & k_{hfd2} \cdot \max(0, \beta - \mathrm{th}) \\ & else\ 0 \end{cases} \quad , IR_I(0) = 1 \ \textbf{(Eq.6)}$$

Where β_f is a variable descrbing the insulin producing capacity of the β cells, σ_{Ibasal} the basal release of insulin, IR_I is a model state describing the change in this capacity during insulin resistance development, σ_I and α_I governs the glucose stimulated insulin release, and k_{clc} is the clearance of insulin. In the description of β_f (**E.q. 5**) the parameter km_β, goverens the sensitivity between insulin secretion and beta cells.

Disease Models & Mechanisms • Supplementary information

In the description of IR_I (**Eq. 6**) k_{hfd2} governs the diet-induced increase in insulin production capacity, and *th* is the same parameter from **Eq. 2** . Lastly, the model description of the fasting plasma glucose was described as:

$$\frac{d}{dt}\mathrm{G} = R0 - EG0 \cdot \mathrm{G} - \frac{\mathrm{S_i}(I^2) \cdot \mathrm{G}}{(I^2 + km_{Si}{}^2) \cdot IR_{Si}}, G(0) = 100 \quad \textbf{(Eq.7)}$$

Where R0 is the basal release of glucose, *EG0* is the basal uptake of glucose, Si and km_{Si} governed the insulin stimulated glucose uptake. Here, IR_{Si} is a model state representing the effect of increased insulin resistance, defined identically as Eq.6, but with a parameter k_{hfd3}.

Parameter values and parameter estimation

The parameter estimation was done using the MEIGO toolbox (3) using the *extend scatter search* (ESS) algorithm (with the dhc local solver option). To get a reasonable start guess we rescaled the Topp et al. model parameters using allometric scaling (from human to mice) and reproducing the same plasma glucose and insulin levels (even for smaller mice beta-cell mass). When running the parameter estimation, we ultimately had to allow for re-estimation of most parameters, however most parameters are still close to the re-scaled Topp et al. values. The following table has all estimated parameter values.

**Parameter Name**	**Unit**	**Estimated (θ_best)**
R0	mg/dL/day	7282
S_i	mL/(uU*day)	378.691
EG0	1/day	14.4945
σ_I	uU/(mL*day)	0.0001
α_I	(mg/dL)^2	18100
k_{clc}	1/day	208.21
Th	(a.u.)	0.54
k_{hfd}	1/day	0.00458527
k_{stz}	(a.u.)	591.72
km_{stz}	mg/kg	1344.26
k_{hfd3}	1/day	0.102156
km_{β}	(a.u.)	0.325071
km_{Si}	uU/mL	113.881
σ_{Ibasal}	uU/(mL*day)	7859.9
k_{hfd2}	1/day	0.0266882
$k_{clc_{stz}}$	L/day	0.02076
V_{stz}	L	0.0175
$k_{a_{stz}}$	1/day	0.6

Disease Models & Mechanisms • Supplementary information

Supplementary References

1. Schmidt H, Jirstrand M. Systems Biology Toolbox for MATLAB: a computational platform for research in systems biology. Bioinformatics. 2006 Feb 15;22(4):514–5. doi:10.1093/bioinformatics/bti799

2. Topp B, Promislow K, deVries G, Miura RM, Finegood DT. A model of beta-cell mass, insulin, and glucose kinetics: pathways to diabetes. J Theor Biol. 2000 Oct 21;206(4):605–19. doi:10.1006/jtbi.2000.2150

3. Egea JA, Henriques D, Cokelaer T, Villaverde AF, MacNamara A, Danciu DP, et al. MEIGO: an open-source software suite based on metaheuristics for global optimization in systems biology and bioinformatics. BMC Bioinformatics. 2014 May 10;15(1):136. doi:10.1186/1471-2105-15-136

Disease Models & Mechanisms • Supplementary information
